# Design and evaluation of an IPE module at the beginning of professional training in medicine, nursing, and physiotherapy

**DOI:** 10.3205/zma001023

**Published:** 2016-04-29

**Authors:** Lena Zirn, Mirjam Körner, Leonie Luzay, Florian Sandeck, Christa Müller-Fröhlich, Christine Straub, Ulrich Stößel, Waltraud Silbernagel, Julia Fischer

**Affiliations:** 1 Universität Freiburg, Medizinische Psychologie & Soziologie, Freiburg, Germany; 2 Gesundheitsschulen Südwest GmbH, Emmendingen, Germany; 3 Universität Freiburg, Bachelorstudiengang Pflegewissenschaft, Freiburg, Germany; 4 Universität Innsbruck, Politikwissenschaft & Soziologie, Innsbruck, Austria

**Keywords:** Professional role, focus groups, education, interdisciplinary health team, interprofessional relations, students

## Abstract

**Aim: **Interprofessional education (IPE) is a central feature of modern education in the health care professions. Despite this, empirically founded and systematically structured IPE courses are absent from many curricula. To answer the WHO’s call for improved interprofessional collaboration in the health care system, a seminar was designed, implemented and evaluated. The target group consisted of students beginning nursing and medical studies (first and second semesters) and physiotherapy students (first year of training).

The aim was to develop a basic IPE module focusing not only on the demands placed by academia and politics, but also the interests of the target group. This module was evaluated on the basis of the modified four-level Kirkpatrick approach.

**Method:** Based on focus group interviews analyzed qualitatively using Mayring’s content analysis, it was possible to define five learning objectives and develop four practice-oriented modules. The seminar was then implemented and evaluated using written pre- and post-seminar evaluations and group discussions.

**Results:** Analysis confirmed the success of the IPE concept in that the seminar was positively rated by attendees not only in terms of their immediate reactions, but also attitude, knowledge and skills according to Kirkpatrick.

**Conclusion:** In the future, it is intended to offer the IPE module on a permanent basis and assess the competencies acquired in the seminar using observation. Courses to ensure sustained learning outcomes would also be desirable.

## Zusammenfassung

**Zielsetzung: **Interprofessionelle Lehre (IPE) ist ein zentraler Bestandteil moderner Ausbildung in den Gesundheitsberufen. Trotzdem fehlen vielerorts empirisch fundierte und systematisch aufgebaute IPE-Angebote in den entsprechenden Curricula. Um der Forderung der WHO nach verbesserter interprofessioneller Zusammenarbeit im Gesundheitswesen nachzukommen, wurde ein Seminarkonzept konzipiert, implementiert und evaluiert. Zielgruppe waren Studienanfänger der Pflegewissenschaft und Humanmedizin (1. und 2. Semester) sowie Physiotherapieschüler (1. Ausbildungsjahr).

Zielsetzung war, ein IPE-Basismodul zu entwickeln, das sich neben Forderungen aus der Wissenschaft und Politik auch an Interessen der Zielgruppe orientiert und selbiges auf Basis des modifizierten Vier-Ebenen Ansatzes von Kirkpatrick evaluiert.

**Methodik: **Basierend auf Fokusgruppeninterviews, die qualitativ mit Mayrings Inhaltsanalyse ausgewertet wurden, konnten fünf Lernziele und vier praxisorientierte Module entwickelt werden. Anschließend wurde das Seminarkonzept implementiert und mit Hilfe einer schriftlichen prä-post Erhebung sowie Gruppendiskussionen evaluiert.

**Ergebnisse: **Die Auswertung bestätigt den Erfolg des IPE-Konzepts indem das Seminar nach Selbsteinschätzung der Teilnehmenden sowohl auf Ebene der unmittelbaren Reaktionen wie auch der Einstellung, des Wissens und der Fertigkeiten nach Kirkpatrick positiv bewertet wurde.

**Schlussfolgerungen:** Zukünftig sollte das IPE-Modul verstetigt werden und im Seminar erworbene Kompetenzen anhand von Beobachtung überprüft werden. Weiterhin wären Veranstaltungen zur Sicherung eines nachhaltigen Lernerfolgs wünschenswert.

## 1. Introduction

### 1.1. Background

Interprofessional education (IPE) takes place around the world in many different forms. An international overview study published by the WHO [1] illustrates major differences in international priorities: two-thirds of existing IPE units have been reported by the USA, Canada, Great Britain and Northern Ireland. While IPE is taking on increased importance internationally [1], such courses in Germany are often elective and ungraded. Curricular implementation of interprofessional courses is generally difficult due to organizational and statutory constraints. As a result, the academization of the non-medical health professions is less advanced in Germany than in countries such as Great Britain and Sweden. These countries also differentiate themselves from Germany in that greater responsibility is given to the practitioners of these professions, in particular the nursing professions [2]. However, there are already positive instances of IPE in Germany, for example the Robert Bosch Stiftung’s project on interprofessional practice in the health care professions [http://www.bosch-stiftung.de/content/language1/html/44092.asp cited 2015 Dec 3].

Given that teamwork is a central factor for improved efficiency in the provision of health care [3] and is defined as a core competency [4], the National Competency-based Catalogue of Learning Objectives in Undergraduate Medical Education (NKLM) [http://www.nklm.de] has restructured medical study with clearer objectives. In particular, the NKLM emphasizes the relevance of collaborative interprofessional teams. Regulations governing other health professions have for some time now specified that interprofessional cooperation is an important aspect of professional practice. For instance, physiotherapists commit themselves in Section 5 of their professional rules and regulations to collaboration with interprofessional teams [https://www.physio-deutschland.de/fileadmin/data/bund/Dateien_oeffentlich/Beruf_und_Bildung/Ausbildung/ZVK-Verband-Berufsordnung-BroschA5-2012-RZ.pdf], and the Deutsche Pflegerat (German Nursing Council) sets down in Section 2 of its Code of Professional Conduct that professional nurses work together in an interdisciplinary manner with other professional groups [http://www.deutscher-pflegerat.de/Downloads/DPR%20Dokumente/Rahmenberufsordnung.pdf].

So that this can be realized and IP anchored in the professional identities of the different health care professions, educational programs and institutions must foster a positive attitude toward interprofessional collaboration (http://www.careum.ch/web/guest/lancet-report) and combat stereotyping [5]. In addition, roles and responsibilities of all the different professions involved must be clear among team members. Only if roles and responsibilities are defined is it possible to treat patients effectively and in a coordinated manner [6], while at the same time decreasing the number of medical errors [7], [8].

#### 1.2. Teaching project aim

The aim of this project was to design, implement and evaluate an IPE course at the University of Freiburg. The project, *Team-centered interprofessional training and education in medicine, nursing and physiotherapy *(TIPAS), intended to encourage interprofessional knowledge and skills among the three professions in general and, more specifically, enable the three professions to become acquainted with each other.

#### 1.3. Main research questions

To design empirically based courses, the first step should be to create module content, and following its implementation, have the target group evaluate it. The study’s aim gives rise to the following research questions:

- Issue 1: How can an IPE course be designed to meet the needs of potential attendees?
- Issue 2: What influence does the IPE course have on the attitudes and skills of the attendees in regard to interprofessional collaboration?

## 2. Project description

In this section the method of seminar development is elaborated on, followed by a description of the seminar concept as a result of the developmental phase. The teaching of the seminar and the evaluation are also covered.

### 2.1. Design method (Issue 1)

To gather information on needs based on the preferences of the target group concerning IPE [9] and thus define learning objectives, focus groups consisting of representatives from all target groups (medicine, bachelor degree program in nursing and physiotherapy) met in meetings lasting 50 minutes each. The focus groups took place separately according to professional field in June, 2014. The groups were each composed of eight medical students, eight from nursing and seven from physiotherapy and were moderated by a project group member, while a second person took the minutes.

The questions followed a guideline and referred to IPE (e.g. How would you like to learn about interprofessionalism?) and roles and stereotypes (e.g. In your opinion, which roles/responsibilities do doctors/physiotherapists/nurses have in providing health care?). To facilitate transcription, audio recordings were made of the interviews. The focus group interviews yielded a total of 55 pages of transcribed material (167 minutes in total).

The subsequent qualitative content analysis based on Mayring [10] was carried out with MAXQDA. After all 232 statements were paraphrased and generalized, inductive codes were assigned during an initial review; these codes were discussed by two team members regarding meaning. This approach was selected in order to remain as closely tied to the content of the text material as possible. Five inductive codes – awareness of issue, lack of appreciation, need for acceptance, identity of role, curiosity – were identified that reflected the statements made by the participants (see attachment 1 ). These were also defined using inductive subcodes. Assertions about the learning format were categorized deductively along the lines of the specific interview questions about learning format. After doing this, the content of the codes were examined and summarized into a key statement and corresponding learning objectives on the basis of frequently cited subcodes. For example, the seminar objective “spheres of responsibility and roles of different health care professions” was derived from the subcodes “lack of knowledge” (category: awareness of issue) and “knowledge about each other” (category: curiosity). Statements about educational needs corresponding to the category of learning format were also taken into consideration when planning the seminar.

#### 2.2. Strategic approach (Issue 1)

Based on the content analysis, five seminar objectives emerged that are central to the target group in respect to IPE and which should be mastered in the unit addressing knowledge:

1. the concepts of interprofessionalism and multiprofessionalism,
2. advantages of IP for patients and medical team,
3. individual and institutional possibilities for fostering IP,
4. spheres of responsibility and roles of different health care professions, and
5. the motives for choosing a particular career.

Table 1 (Tab. 1) contains explanations of the objectives.

The learning objectives were then transformed into four modules to structure the sequence of the course: introduction, stereotypes, role profiles, and wrap-up (see table 2 (Tab. 2)).

In regard to teaching the course, the majority of participants expressed a desire for a strong focus on practice (six statements were made for centering on practice vs. none for centering on theory) and scheduling the seminar early on in the curriculum (nine statements for early inclusion vs. six for later inclusion). This corresponds with the recommendations of Anderson & Thorpe [11] who advocate anchoring IPE early on in education as a basis for future interprofessional collaboration in day-to-day professional practice.

#### 2.3. Method for seminar evaluation (Issue 2)

In keeping with the recommendations of the Institute of Medicine [12], the evaluation of the seminar followed the mixed-methods approach and was designed according to the first two dimensions of Kirkpatrick’s four-level approach [13] as modified by Barr, Koppel, Reeves, Hammick & Freeth [14].

The level-1 reaction (Did they enjoy it?) was assessed by holding a group discussion at the end of the class session. In addition, the relevance of IPE was assessed by the item in the questionnaire asking how important students considered interprofessional courses to be on an eleven-point Likert scale (0=not at all to 10=very important). Satisfaction was measured after the seminar using seven eleven-point items (0%= does not apply at all to 100%=fully applies). An example being the item, “I would recommend this seminar to others.” For all of the items reported here, refer to the post-seminar evaluation questionnaire in attachment 2 .

To gather insights on levels 2a) attitude and reaction and 2b) knowledge and skill, a survey instrument was developed and filled out by the attendees both before and after the seminar session (pre-seminar evaluation: online prior to the seminar; post-seminar evaluation: immediately after the seminar). The pre-seminar version consisted of six sociodemographic items (age, sex, prior experience, and current training/study program) and 22 items representing knowledge and attitude regarding IPE. Nine of these items referred to the seminar objectives aimed for and are evaluated in the following. These internally developed items are formulated as statements, for instance, “I know the advantages of interprofessional collaboration,” and each statement is rated on a five-point Likert scale (1=does not apply at all to 5=fully applies). To gather information on previous experience with interprofessional collaboration, space was left for open-ended responses with instructions to “please describe the characteristics of successful interprofessional teamwork in health care.” The post-seminar version of the questionnaire was reviewed and adapted after the first TIPAS seminar during the winter semester 2014/15, a process that led to the deletion of six items.

#### 2.4. Seminar implementation (Issue 2)

After designing the seminar, it was offered for two consecutive semesters (winter 2014/15 and summer 2015), both times as a seven-hour seminar block. To establish a shared knowledge base [12], the registered attendees received reading material in advance after submitting the pre-seminar evaluation questionnaire (see 2.3). The concepts of interprofessionalism and multiprofessionalism were differentiated from each other and one-page long educational profiles of each of the three professions – medicine, physiotherapy, and nursing – were presented. A common knowledge base is particularly critical since the course is for those who are only first starting their vocational training or university study and have not yet had the chance to develop a clear understanding of the different roles, including their own. 

Each of the professions was represented by one experienced practitioner; additionally an instructor with a specialty in interprofessionalism and teamwork was on the teaching team, as required by the Institute of Medicine [15]. Thirty-five university students and vocational students in medicine, physiotherapy and nursing attended the seminar, with four registered participants who dropped out. Of the total number, 15 were medical students (first or second semester), 12 were students in the bachelor degree program in nursing (first to fourth semester and simultaneously second to third year of vocational nursing training), and eight physiotherapy students (first to second year of training). The mean age was 23 years (min: 18; max: 40; SD: 4.83), and the relationship between the sexes with 73.7% female students reflected that of the student cohorts. Attendance was on a voluntary basis for all participants. While the university students in medicine or nursing received academic credit for the seminar, the physiotherapy students received certificates of attendance upon completing the course.

#### 2.5. Seminar evaluation (Issue 2)

Quantitative data analysis was carried out on the entire data set of 35 attendees from the first (*n*=15) and second (*n*=20) seminar cycles. Due to the small sample size and absence of a normal distribution, the Wilcoxon rank-sum test was applied to determine significant changes in knowledge and attitudes concerning interprofessionalism as a result of the seminar.

##### 2.5.1. Level 1: Reactions

The importance of interprofessional courses was ranked highly before the seminar (*M*=8.68) and significantly higher after the seminar (*M*=9.35), *z*=-2.99, *p*=.003, *r*=-.51. In the post-seminar evaluation there was a high level of satisfaction among attendees with mean values between 8.26 (*SD*=2.21) for the item “My attitude toward interprofessional teamwork was positively affected by the seminar” and 9.30 (*SD*=1.07) for the item “I would recommend the seminar to others” (see table 3 (Tab. 3)).

The group discussion after the seminar was recorded and the content was then assigned to the main aspects of the learning objectives. In respect to pointing out personal and institutional possibilities for fostering IP (sub-goal 3), the physiotherapy students commented that the seminar helps to raise awareness of stereotyping. Nursing attendees agreed with this assessment and added that stereotyping should be avoided, while several medical students stated that stereotypes help make it easier to identify and assign the tasks to the professional groups. In respect to the advantages of IP for patients and medical teams (sub-goal 2), the nursing students said that they felt encouraged to bring up problems when working in interprofessional teams and to actively request assistance when they needed it. Medical students added that the advantages of interprofessional collaboration for patients became clear, even if this required some effort. They drew this insight primarily from the experience with the case vignette, in which it was the nursing students in particular who were able to share detailed knowledge since they had prior practical experience in providing patient care. This is why the course participants discussed whether or not it would make more sense if the seminar were offered at a later point in the curriculum when all of the participants would have acquired some prior practical experience. Knowledge about the roles and responsibilities of the different health care professions (sub-goal 4) was viewed as being improved after the seminar. In this context, grappling with self-image and the images of the other professional groups in the group exercises was responded to positively.

##### 2.5.2. Level 2: Attitudes, knowledge and skills

As shown in table 4 (Tab. 4), the mean values for the pre-seminar evaluation of the selected items lay between 2.29 (*SD*=0.91) and 3.97 (*SD*=0.76). After the seminar these values lay between 4.09 (*SD*=0.57) and 4.76 (*SD*=0.44). In respect to the seminar objectives there was a significant improvement in the rating of these items in terms of the concepts of interprofessionalism and multiprofessionalism [1], the advantages of IP for patients and medical teams [2], personal and institutional means to foster IP [3], roles and responsibilities of different health care professions [4], and motives for choosing a particular career path [5].

## 3. Discussion

As a result of the needs-based approach using focus group analysis, a seminar concept appropriate to the target group was drafted. At the same time, this seminar strategy aligned with current developments and standards, such as addressing professional profiles and roles [5], [9], [10], [16]. From the five focus group objectives, four modules were designed that imparted both theory and practical experience. An interprofessional instructor team taught these modules to an interprofessional group of seminar attendees (Issue 1). An inductive approach using focus groups was selected as a way to give consideration to the preferences of potential seminar participants regarding content and design. Measured by the benefits to seminar design, this approach can be viewed as adequate since it was possible to identify relevant seminar topics using the focus groups. Other topics specific to the target group were taken into account, such as covering foreign health care systems and familiarization with the educational curricula of other health care professions. The quality of the evaluation of the focus groups must, however, be viewed as limited. For lack of capacity, no triangulation or communicative validation was carried out, and no data on inter-rater reliability was collected.

The results of the evaluation and the reactions to the seminar were very positive (Issue 2). Knowledge was acquired on all five seminar goals, while at the same time the attitude toward the relevancy of IPE was generally strengthened. The positive ratings for the seminar indicate a purposeful and interest-oriented seminar concept, as well as effective implementation. Significant improvements for the knowledge items on the questionnaire, however, do not allow any assertions about an actual improvement in skills at the behavior level, but do reflect subjective competency expectations of the seminar attendees. Bandura [13] argues in his self-efficacy theory that the expectation of being able to perform a certain personal or professional task correlates with the actual performance of that task. In contrast, there are studies in the field of medical education whose results were unable to find a connection between subjective assessment and objective performance [14], [16]. Still, it can be assumed that high subjective expectations of abilities positively affect motivation and willingness to apply what has been learned [13].

Offering the seminar at the beginning of the curriculum was favored by the majority of the focus group participants and follows the recommendations of Areskog [17] to combat stereotyping early on. During the seminar, particularly while working on the interprofessional case work, a gap in knowledge and skills became apparent between medical students, nursing students and physiotherapy students, since the nursing students had already had the opportunity to gather practical experience. Carpenter [5] cites multiple studies that show IPE participants must first have basic competency and feel confident in their own professional role before they can participate in interprofessional learning. This discrepancy was also visible in this project. The seminar should be practice-centered and professionally relevant competencies should be fostered, but the case material should not require too much medical expertise. Still, it must be stated that it was particularly this work on the interprofessional case vignettes that was rated so positively as a learning experience by the seminar attendees.

### 3.1. Limitations

The seminar and the evaluations were included in the regular semester course load for the three professions and were subject to organizational constraints. The field study nature of this investigation also leads to limitations regarding quality criteria. That being said, the feasibility of this approach for future years can still be evaluated positively.

#### 3.1.1. Design

Due to the small sample size and the short duration of the course, one day in each case, the validity of the study results must be viewed as limited. The effort of teaching is high with four instructors and a maximum of 18 student participants per course. On the other hand, demonstrating in person the principles which the course seeks to impart is indispensible for the success of IPE [18]. The interprofessional instructor team in this case was exemplary in modeling collaborative behavior. Also, this format for introducing interprofessional issues is more popular with first and second-semester students in the health care professions than lectures on the same topic [19]. The small amount of previous experience, the defined learning objectives and the single-day length of the seminar make valid measurements of collaborative skills as called for by Kirkpatrick [20] und Barr et al. [21] impossible. Only knowledge and attitudes could be captured on the questionnaires, but not skills or behavior [22]. Despite this, the seminar consisted of many group exercises in which the participants applied the communicative strategies aimed for and were able to assume their specific professional roles. Accordingly, this course was competency-based, while the evaluation was knowledge-based.

##### 3.1.2. Implementation and evaluation

The small sample size was further reduced by the drop out of physiotherapy students: during the first course offering only three of the six registered students showed up. In response, an announcement was made prior to holding the seminar for the second time that attendance certificates would be issued, leading to a reduction in the drop-out rate.

Furthermore, a selection bias must be assumed since the seminar participants attended it voluntarily for the most part: a student interested in IPE would need to show a great degree of motivation to attend the seminar. For this reason, the reported results can be applied only generally to the total university and vocational student populations in the health care professions.

Due to the small sample size so far, psychometric verification of the measuring instrument has not yet been possible, the reason why the questionnaire does not represent a valid evaluation of achieving the seminar goals. Still, the high values for satisfaction (see table 3 (Tab. 3)) show that the seminar is accepted and considered valuable by the attendees.

#### 3.2. Outlook

The strategy presented here has been confirmed in practice, and the seminar will continue to be offered at least once a year. As a topical supplement, an advanced module on the subject of interprofessional communication will take place. In order to make any claims about the acquisition of skills, an instructor guideline on making observations is being planned so that it will be possible to evaluate the work done on the interprofessional case vignettes. In general, the benefits of counting the course toward study credit should be regulated in the same manner for all the groups to combat any systematic drop outs. Courses or events to ensure sustained learning outcomes would also be desirable [23]. In addition, implementation in the curriculum could open the seminar up to a larger number of university and vocational students and also enable psychometric testing of the questionnaire.

## Competing interests

The authors declare that they have no competing interests.

## Supplementary Material

Table A-1. Description of codes and subcodes for the evaluation of focus group interviews (along with quotes as examples)

Post-questionnaire (shortened version)

## Figures and Tables

**Table 1:**
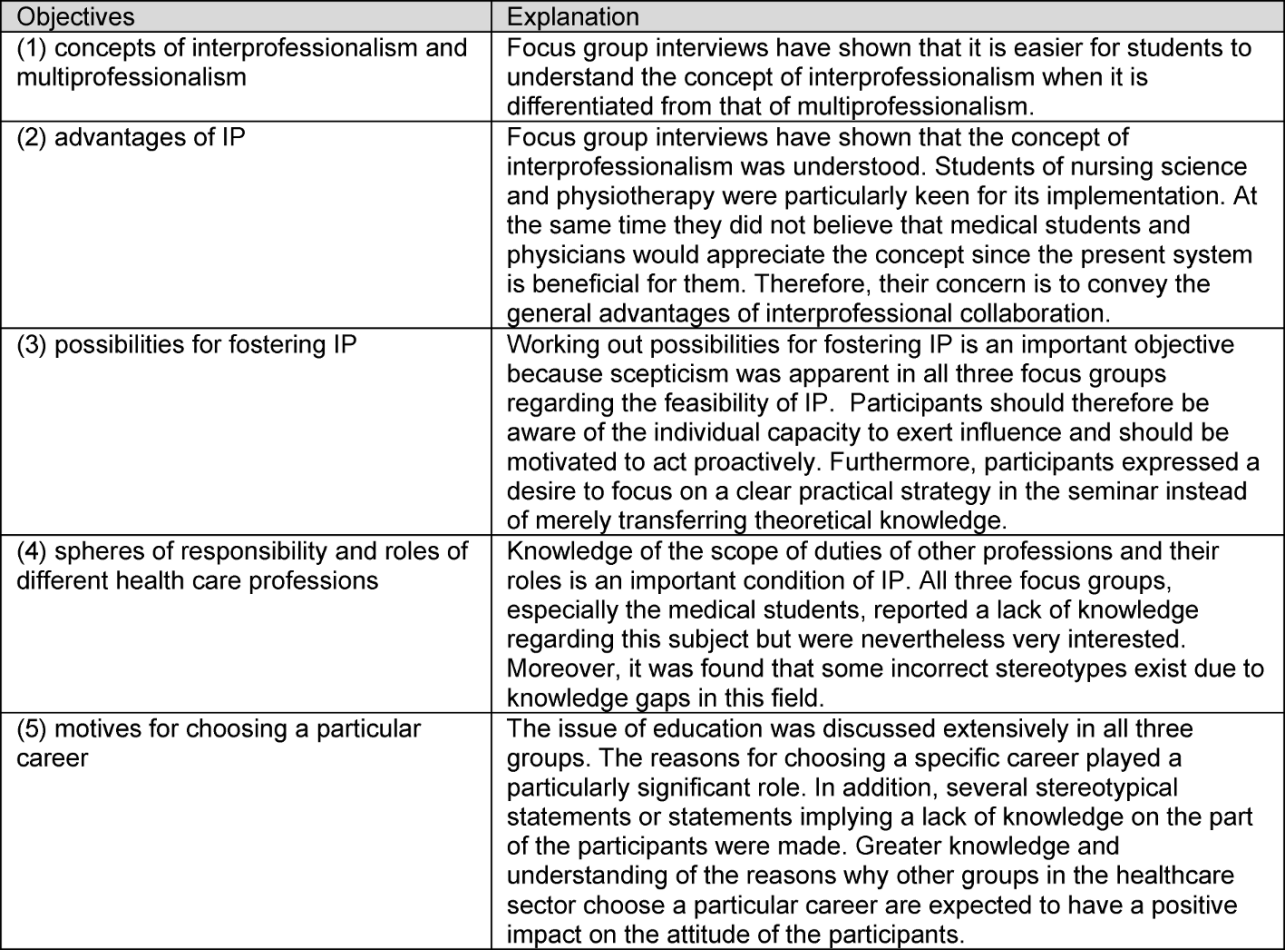
Explanation of the five objectives resulting from the focus groups.

**Table 2:**
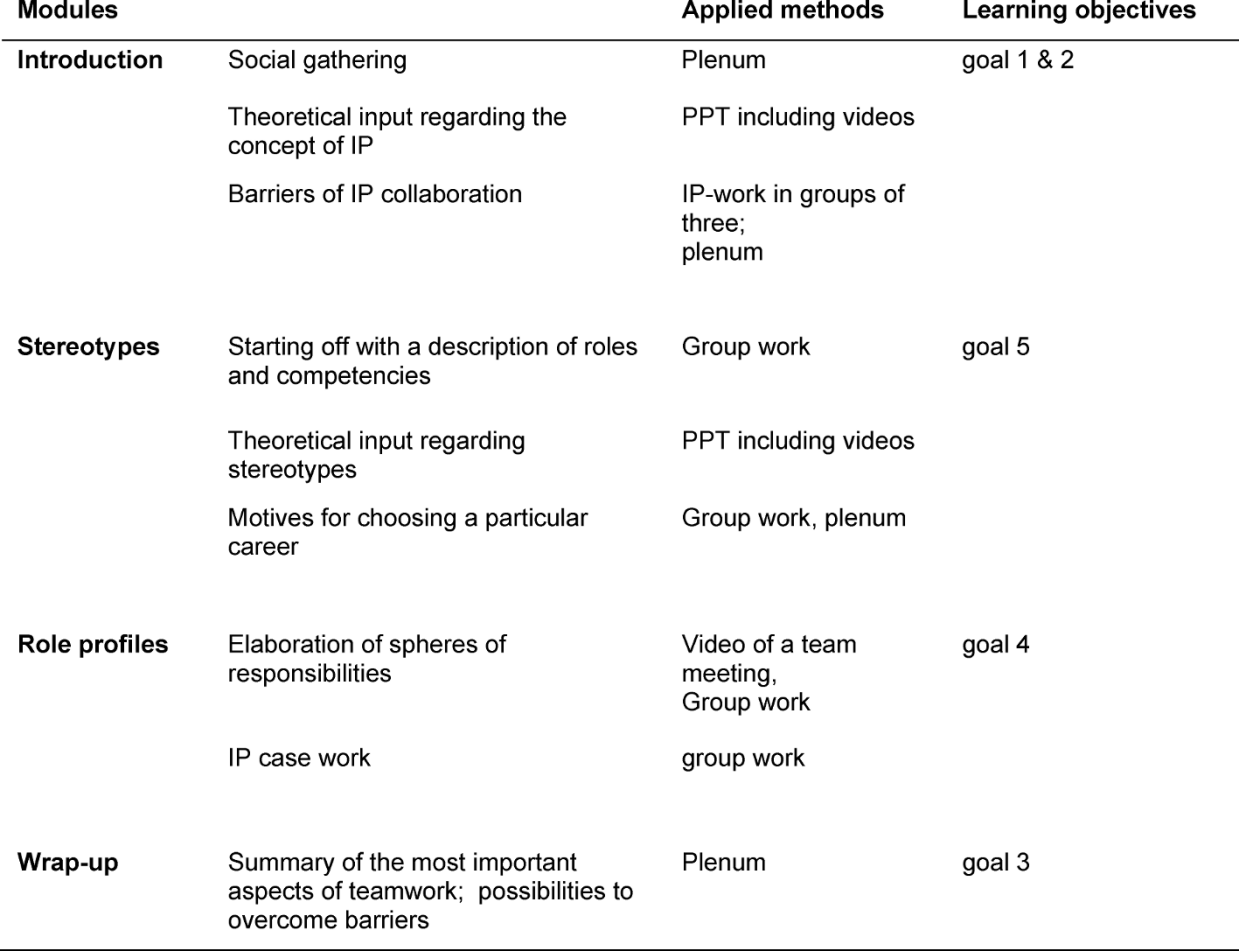
Overview of the seminar content, the applied teaching methods and learning objectives

**Table 3:**
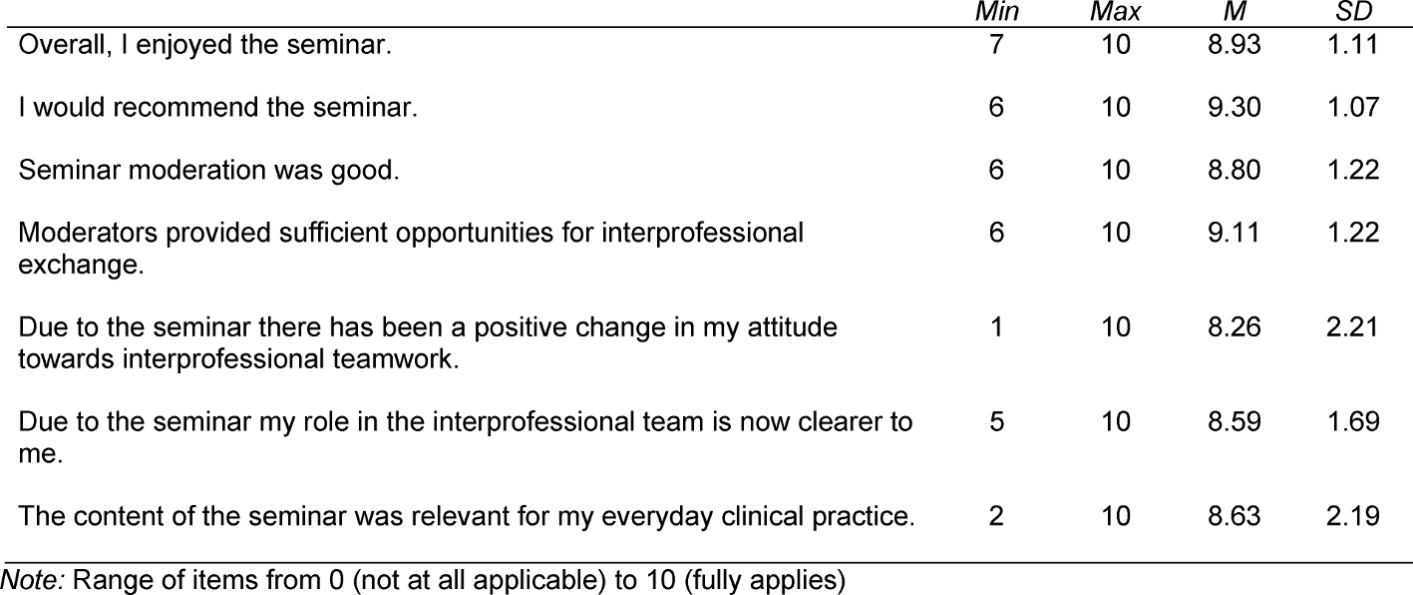
Descriptive Statistics of items regarding satisfaction

**Table 4:**
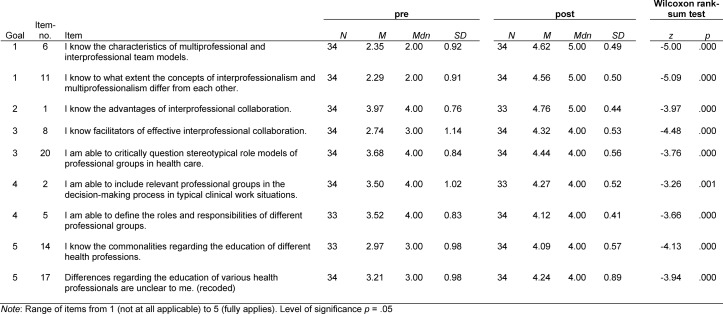
Pre-Post comparison of selected items regarding the seminar‘s learning objectives
