# Supplementary material for: Design and evaluation of an IPE module at the beginning of professional training in medicine, nursing, and physiotherapy
Source: GMS J Med Educ. 2016 Apr 29;33(2):Doc24. doi: 10.3205/zma001023 (PMC4895859; doi:10.3205/zma001023)
Supplement: Table A-1. Description of codes and subcodes for the evaluation of focus group interviews (along with quotes as examples) [file JME-33-24-s-001.pdf]

Code	Subcode	Key message	Exemplary quote
**Awareness of issue**<br>*This code reflects statements which refer to barriers of interprofessionalism.*	Interface communication	Criticism of effectiveness and information loss in interface communication at the expense of patients	„… there are probably physicians who work for over 20 years in a clinic and who are happy with hierarchy - based on the motto that's my job and the rest is yours - and then it's difficult, of course, to convince everybody to try interprofessionalism …“ [medicine – 00:06]
	Mediating role	Nurses are considered as intermediaries between different health professions working in the clinic.	
	Issue of prejudices	It was postulated that both health professionals *per se* and society have prejudices against different health professions.	
	Health system factors	Existing structures of the German health system (e.g. the legal framework of professional competencies and the heavy workload of persons affected) are believed to hinder interprofessionalism.	
	Division of labour	Consequences of the distribution of tasks such as repetitive work procedures (e.g. establishing medical history) and competing expertise are regarded as inefficient and problematic.	
	Lack of knowledge	Some professional groups know too little about each other and respective responsibilities. Greater respective knowledge would be invaluable because professions could benefit from each other's expertise.	
	Entrenched structures	There are entrenched structures in the health system; it is especially difficult to convince elderly doctors of the benefits of interprofessionalism.	
**Lack of appreciation**<br>*This code includes statements which reflect a lack of appreciation of other health professional groups.*	Motives for choosing a particular career	Assumptions why different people choose a particular career.	" This is a malicious allegation regarding physicians but some probably do it because of social status" [physio – 00:48]
	Hierarchy	Hierarchy between professional groups was mentioned as a barrier for interprofessional work.	
	Competencies	Statements by participants in different professional groups that the qualifications and competencies of another professional group are minor.	
	Soft Skills	Doctors are considered to have fewer social skills and are described as arrogant.	

**Need for acceptance**<br>*This code reflects statements that stress the need for acceptance and appreciation of other professional groups.*	Training programs	Degrees and training options that qualify towards practicing a profession are not equally accepted	"One main difference is that doctors complete an academic education whilst nurses and physiotherapists undergo normal training. Therefore, doctors might feel special or better qualified …" [nurse – 00:24]
	Professional competence	Lack of recognition by other professionals regarding nurses' professional competencies.	
	Equivalence of labour	The work of physiotherapists and nurses is not considered to be equivalent to the work of physicians.	
**Identity of role**<br>*This code reflects statements that prove the existence of a sense of belonging to one's own professional group.*	Remit	Statements of individual professional groups about the relevance and scope of their remit.	"Physios have a wide range of interests. Post- training you can work in so many different areas. " [Physio - 00:45:37]
	Qualification	Statements by individual professional groups which clearly show that they consider themselves as qualified.	
	Understanding of roles	Statements regarding the own role in interprofessional teamwork.	
**Curiosity**<br>*This code reflects statements that show particular interest in the content of the one-day workshop.*	Knowledge about each other	Request to learn about the specific remits of other professions and to establish how the task domains can be differentiated from each other.	"I think it's really important to provide information so that everybody really knows what others do. I believe that this would promote understanding and possibly improve the basis for more effective communication. " [nurse – 00:10]
	Commonalities and strength	Request to focus on commonalties and strengths instead of differences.	
	interprofessionalism	The seminar shall demonstrate the advantages of interprofessionalism for patients and therapists.	
	Casework and practical relevance	The seminar shall be practice-orientated involving example cases and practical exercises.	
	Seating arrangements	Nurses und physios recommend a seating arrangement so that the participants work via an interdisciplinary approach.	
	Joint learning	The proposal to offer joint courses, e.g. lectures, when teaching content is redundant.	
	Interprofessional comparison	Request to look at interprofessional teamwork in other countries.	
