# Supplementary material for: Design and evaluation of an IPE module at the beginning of professional training in medicine, nursing, and physiotherapy
Source: GMS J Med Educ. 2016 Apr 29;33(2):Doc24. doi: 10.3205/zma001023 (PMC4895859; doi:10.3205/zma001023)
Supplement: Post-questionnaire (shortened version) [file JME-33-24-s-002.pdf]

Post-questionnaire (shortened version)

.		Does not apply at all	Unlikely to apply	Don’t know	Likely to apply	Fully applies
		**1**	**2**	**3**	**4**	**5**
1	I know the advantages of interprofessional collaboration.	☐	☐	☐	☐	☐
2	I am able to include relevant professional groups in the decision-making process in typical clinical work situations.	☐	☐	☐	☐	☐
5	I am able to define the roles and responsibilities of different professional groups.	☐	☐	☐	☐	☐
6	I know the characteristics of multiprofessional and interprofessional team models.	☐	☐	☐	☐	☐
8	I know facilitators of effective interprofessional collaboration.	☐	☐	☐	☐	☐
11	I know to what extent the concepts of interprofessionalism and multiprofessionalism differ from each other.	☐	☐	☐	☐	☐
14	I know the commonalities regarding the education of different health professions.	☐	☐	☐	☐	☐
17	Differences regarding the education of various health professions are unclear to me.	☐	☐	☐	☐	☐
20	I am able to critically question stereotypical role models of professional groups in health care.	☐	☐	☐	☐	☐

Please describe the characteristics of successful interprofessional teamwork in health care

How important do you consider interprofessional courses to be? Allocate a score between **0 (not at all) and 10 (very important)**

0	1	2	3	4	5	6	7	8	9	10
☐	☐	☐	☐	☐	☐	☐	☐	☐	☐	☐

	Does not apply at all										Fully applies
	0%	10%	20%	30%	40%	50%	60%	70%	80%	90%	100%
1. Overall, I enjoyed the seminar.	☐	☐	☐	☐	☐	☐	☐	☐	☐	☐	☐
2. I would recommend the seminar.	☐	☐	☐	☐	☐	☐	☐	☐	☐	☐	☐
3. Seminar moderation was good.	☐	☐	☐	☐	☐	☐	☐	☐	☐	☐	☐
4. Moderators provided sufficient opportunities for interprofessional exchange.	☐	☐	☐	☐	☐	☐	☐	☐	☐	☐	☐
5. Due to the seminar there has been a positive change in my attitude towards interprofessional teamwork.	☐	☐	☐	☐	☐	☐	☐	☐	☐	☐	☐
6. Due to the seminar my role in the interprofessional team is now clearer to me.	☐	☐	☐	☐	☐	☐	☐	☐	☐	☐	☐
7. The content of the seminar was relevant for my everyday clinical practice.	☐	☐	☐	☐	☐	☐	☐	☐	☐	☐	☐
